# Dietary Phosphorus Levels Influence Protein-Derived Uremic Toxin Production in Nephrectomized Male Rats

**DOI:** 10.3390/nu16121807

**Published:** 2024-06-08

**Authors:** Dennis P. Cladis, Kendal M. Burstad, Annabel Biruete, Amber H. Jannasch, Bruce R. Cooper, Kathleen M. Hill Gallant

**Affiliations:** 1 Department of Food Science and Nutrition, University of Minnesota, St. Paul, MN 55108, USA; dcladis@vt.edu (D.P.C.); schm3950@umn.edu (K.M.B.); 2 Department of Nutrition Science, Purdue University, West Lafayette, IN 47907, USA; abiruete@purdue.edu; 3 Department of Food Science and Technology, Virginia Polytechnic Institute and State University, Blacksburg, VA 24060, USA; 4 Department of Nutrition and Dietetics, Indiana University-Purdue University Indianapolis, Indianapolis, IN 46202, USA; 5 Division of Nephrology, Indiana University School of Medicine, Indianapolis, IN 46202, USA; 6 Bindley Bioscience Center, Purdue University, West Lafayette, IN 47906, USA; hopfas@purdue.edu (A.H.J.); brcooper@purdue.edu (B.R.C.)

**Keywords:** uremic toxins, chronic kidney disease (CKD), phosphorus, gut microbiota

## Abstract

Gut microbiota-derived uremic toxins (UT) accumulate in patients with chronic kidney disease (CKD). Dietary phosphorus and protein restriction are common in CKD treatment, but the relationship between dietary phosphorus, a key nutrient for the gut microbiota, and protein-derived UT is poorly studied. Thus, we explored the relationship between dietary phosphorus and serum UT in CKD rats. For this exploratory study, we used serum samples from a larger study on the effects of dietary phosphorus on intestinal phosphorus absorption in nephrectomized (Nx, n = 22) or sham-operated (sham, n = 18) male Sprague Dawley rats. Rats were randomized to diet treatment groups of low or high phosphorus (0.1% or 1.2% *w*/*w*, respectively) for 1 week, with serum trimethylamine oxide (TMAO), indoxyl sulfate (IS), and p-cresol sulfate (pCS) analyzed by LC-MS. Nx rats had significantly higher levels of serum TMAO, IS, and pCS compared to sham rats (all *p* < 0.0001). IS showed a significant interaction between diet and CKD status, where serum IS was higher with the high-phosphorus diet in both Nx and sham rats, but to a greater extent in the Nx rats. Serum TMAO (*p* = 0.24) and pCS (*p* = 0.34) were not affected by dietary phosphorus levels. High dietary phosphorus intake for 1 week results in higher serum IS in both Nx and sham rats. The results of this exploratory study indicate that reducing dietary phosphorus intake in CKD may have beneficial effects on UT accumulation.

## 1. Introduction

Chronic kidney disease (CKD) affects 15% of US adults and significantly increases the risk of morbidity and mortality due to declining kidney function [1]. Because there is no cure for CKD, the primary goal of treatment is slowing disease progression and preventing kidney failure. To meet this goal, the cornerstone of CKD treatment is diet alteration, with much work investigating the effects of various dietary components—including phosphorus (P), calcium (Ca), and protein—and their impacts on kidney health [2]. As kidney function declines, mineral metabolism becomes increasingly disordered, leading to arterial calcification and the development of chronic diseases including cardiovascular disease (CVD) and bone diseases [3,4].

Preclinical and clinical studies report that alterations in mineral metabolism negatively affect the gut–kidney axis in CKD, giving rise to a dysbiotic gut microbiota that increases the production of uremic toxins (UT) [5,6,7]. As kidney function declines, renal excretion of UT via tubular secretion diminishes [8]. This combination of increased UT production and decreased renal excretion causes UT to accumulate in the circulation of CKD patients, which may cause further impairment of kidney function and damage other organ systems such as the cardiovascular system, bone, and liver [9].

**Figure 1:**
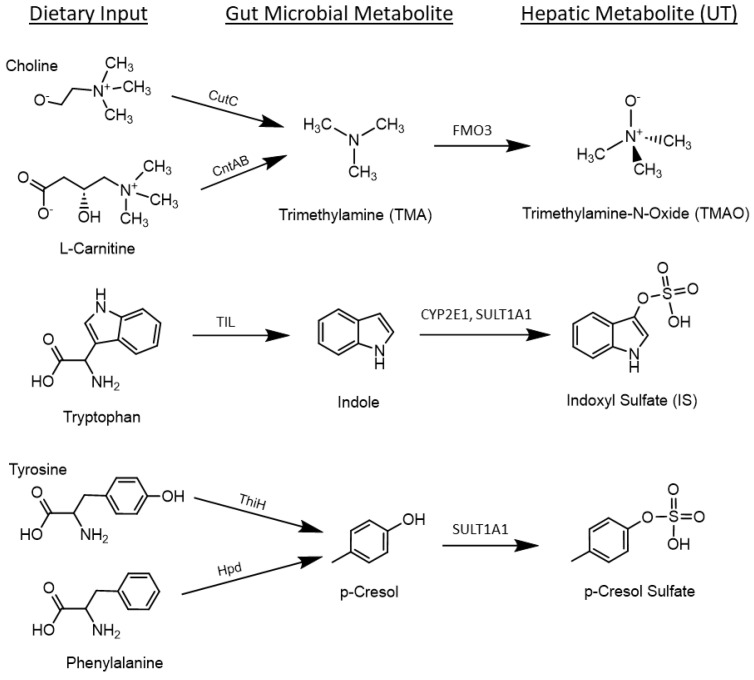
Formation of the uremic toxins (UT) trimethylamine-N-oxide (TMAO), indoxyl sulfate (IS), and p-cresol sulfate (pCS). Dietary choline, L-carnitine, tryptophan, tyrosine, and phenylalanine are metabolized by the gut microbiota to form trimethylamine (TMA), indole, and p-cresol. These precursor molecules are absorbed and transported to the liver where they are further metabolized by hepatic enzymes to form UT. (CutC = choline trimethylamine-lyase; CntAB = carnitine monooxygenase; CYP2E1 = cytochrome P450 family 2 subfamily E member 1; FMO3 = flavin-containing monooxygenase 3; Hpd = p-hydroxyphenylacetate decarboxylase; SULT1A1 = sulfotransferase family 1A member 1; ThiH = tyrosine lyase; TIL = tryptophanase (tryptophane indole-lyase)).

Gut-microbially generated UT are derived from dietary proteins, with many reports detailing the effect of different dietary protein levels on the production and retention of UT in CKD [5,10,11]. Though many microbially derived UT have been identified, the most frequently studied are trimethylamine N-oxide (TMAO), indoxyl sulfate (IS), and p-cresol sulfate (pCS) because they are elevated in patients with CKD and cause the acceleration of CKD progression and comorbidities like cardiovascular disease [12,13,14]. TMAO, IS, and pCS are metabolites of dietary choline/L-carnitine, tryptophan, and tyrosine/phenylalanine, respectively (Figure 1) [15,16]. These metabolites are formed in a two-step process, whereby the gut microbiota metabolizes these dietary inputs to UT precursors (TMA, indole, and p-cresol) that are absorbed and then further metabolized into their final form (TMAO, IS, and pCS) via microbial and hepatic metabolism [5,10,16,17].

Because there is no cure for CKD, altering dietary patterns is the cornerstone of treatment. Current clinical nutrition guidelines for patients with moderate CKD (i.e., stage 3–5 not on dialysis) recommend both low protein intake and reducing dietary phosphorus intake [18]. In the context of UT, the primary research focus has been the relationship between dietary protein and the production of UT, with many reports of a direct relationship between dietary protein intake, serum UT levels, and adverse health outcomes [5,10,11]. Interestingly, many of the microbial organisms in the gut microbiome responsible for the metabolism of protein to UT rely on phosphorus as a key nutrient for cellular processes, though the impact of dietary phosphorus intake on UT formation is currently unknown. Phosphorus is a key element for the survival and growth of the gut microbiota and recent studies have shown that alterations to phosphorus with dietary interventions or the use of phosphate binders can substantially alter the composition of the gut microbiota [19,20,21]. Therefore, we hypothesized that dietary phosphorus restriction may lead to altered serum UT levels. We explored this by evaluating the effect of dietary phosphorus restriction on serum UT levels in 5/6th nephrectomized (Nx) rats as a model for CKD, and sham-operated rats as healthy comparisons.

## 2. Materials and Methods

### 2.1. Study Design

This study is a secondary analysis of a larger study evaluating the effects of dietary phosphorus on intestinal phosphorus absorption. The full study design and details are presented elsewhere [22]; here, we present a summary of the parent study with details relevant to the secondary analysis reported herein.

The parent study employed a 2 × 3 factorial design (factors = health status and dietary phosphorus load) using n = 72 commercially available Sprague Dawley rats (Charles River, Indianapolis, IN, USA). Eight-week-old male rats underwent either a 5/6th nephrectomy (Nx, n = 36) or sham operation (n = 36) at Charles River prior to shipment to Purdue University ~1 week post-surgery. Rats were received in four shipment cohorts (n = 18/shipment), with each shipment containing n = 9 Nx and n = 9 sham-operated rats. Rats were randomized to one of three dietary treatments within the four shipment blocks and the two disease states, using a complete block design. During a 3 week acclimatization period, investigators handling the rats were blinded to group allocation. Upon arrival, rats were pair-housed in solid-bottom cages with Aspen bedding on a 12 h light/dark cycle in a temperature- and humidity-controlled room. After 2 weeks, rats were transferred to wire-bottom cages and individually housed for 1 week prior to starting diet treatment; rats remained individually housed in wire-bottom cages until the end of the study.

During the 3 week acclimatization period, rats were maintained on the Teklad 2018 non-autoclaved rodent diet (Envigo, Indianapolis, IN, USA). This diet contained 0.7% phosphorus (*w*/*w*) and was fed ad libitum for 3 weeks to allow for the development of kidney disease. At the end of this period (i.e., 4 weeks post-surgery and ~12 weeks of age), rats were treated for 1 additional week with one of three treatment diets: low phosphorus (low P; 0.1% *w*/*w* [Teklad TD.85010]), high phosphorus (high P; 1.2% *w*/*w* [Teklad TD.85349]), or low phosphorus followed by acute high phosphorus on the last (7th) day (LPHP). (For this secondary analysis, LPHP rats were excluded from analysis because rats were only on the high P diet for 4 h and euthanized 2 h later, making it unlikely that the lower intestinally generated UT precursors would have reached peak levels. Thus, to prevent potential confounding in this exploratory study, the a priori decision was made to only analyze data from animals that had been on the low P or high P diets consistently for 1 week.)

The dietary phosphorus levels and treatment groups were selected based upon a previous study by Giral et al. [23]. The high P and low P diets were similar in nutrient content to the Teklad 2018 diet, though the nutrient sources were different. In Teklad 2018, the primary ingredients were wheat, corn, wheat middlings, soybean meal, corn gluten meal, and soy oil, while the primary ingredients for the high P and low P diets were sucrose, egg white solids, corn starch, corn oil, and cellulose. Non-phytate phosphorus, which has significantly higher bioavailability than phytate phosphorus [24], was present in the diets at 0.1%, 0.4%, and 1.2% (*w*/*w*) in the low P, Teklad 2018, and high P diets, respectively. (For a detailed description of the study diets, see Tables S1 and S2). During the 1 week treatment period, access to food was restricted to a daily 4 h feeding window, with water available ad libitum. The restricted feeding protocol was required by the parent study to determine the pharmacokinetics of phosphorus absorption [22].

Upon completion of the 4 h feeding window on the last day of the study (i.e., 7th day), radiolabeled ^33^P was administered via oral gavage and phosphorus pharmacokinetics assessed via periodic blood draws over 2 h. Upon completion, rats were euthanized via CO_2_ asphyxiation. Blood draws for assessment of UT and kidney function were taken at baseline (i.e., day 1, before the start of treatment diets; jugular vein draw) and after euthanasia (i.e., day 7; abdominal aortic draw). Blood samples were immediately aliquoted into both uncoated tubes and lithium heparin-coated tubes for serum and plasma collection, respectively (Sarstedt, Numbrecht, Germany). Serum and plasma were stored at −80 °C until analysis. At necropsy, kidneys were inspected to confirm sham or Nx operation: all sham-operated rats had two fully intact kidneys, while Nx rats were missing the right kidney and only had the central ~1/3 of the left kidney present. 

Rat experiments were conducted in adherence with Purdue University Animal Care and Use Committee (PACUC) guidelines, following an approved protocol (1402001030) that ensured the 3Rs of animal research are followed (i.e., replacement, reduction, and refinement). This study protocol was pre-registered at Animal Study Registry [25].

### 2.2. Analytical Methods

#### 2.2.1. Blood Urea Nitrogen (BUN)

Plasma samples collected at sacrifice were analyzed for blood urea nitrogen (BUN) by a commercial colorimetric kit (Urea Assay Kit, BioAssay Systems, Hayward, CA, USA) following the manufacturer’s instructions.

#### 2.2.2. Uremic Toxins (UT)

Serum samples collected at sacrifice in all rats (n = 7–11/group) and serum samples collected at baseline from a subset of rats (n = 3–4/group) were analyzed for TMAO, IS, and pCS. Collected serum samples were frozen at −80 °C immediately after collection and stored for two months until analysis in this study. Samples were extracted by mixing 50 µL serum with 150 µL ice-cold methanol (MeOH) and 10 µL of a mixture of deuterated UT analogs containing 33 ng/µL IS-d4, 33 ng/µL pCS-d7, and 166 ng/µL TMAO-d9. Samples were vortex mixed for 10 min then separated via centrifugation for 10 min at 16,200× *g*. The supernatant was separated, dried, and resuspended in 50 µL of a 95:5 mixture of water–acetonitrile with 0.1% formic acid.

An Agilent 1290 Infinity II liquid chromatography (LC) system coupled to an Agilent 6470 series QQQ mass spectrometer (MS, Agilent Technologies, Santa Clara, CA, USA) was used to analyze UT metabolites. A Water’s Corporation HSS T3 2.1 mm × 150 mm, 1.8 µm column was used for LC separation (Water’s Corporation, Milford, MA, USA). The buffers were (A) water + 0.1% formic acid and (B) acetonitrile + 0.1% formic acid. The linear LC gradient was as follows: time 0 min, 0% B; time 2 min, 0% B; time 6 min, 100% B; time 7 min, 100% B; time 7.1 min, 0% B; time 10 min, 0% B. The flow rate was 0.3 mL/min. Multiple reaction monitoring was used for MS analysis. The data were acquired in negative and positive electrospray ionization (ESI) mode (see Table S3 for details). The jet stream ESI interface had a gas temperature of 325 °C, gas flow rate of 8 L/min, nebulizer pressure of 45 psi, sheath gas temperature of 250 °C, sheath gas flow rate of 7 L/min, capillary voltage of 3500 V in positive mode and negative mode, and nozzle voltage of 1000 V. The Δ electron multiplier voltage (ΔEMV) was 300 V in both positive and negative modes. All data were analyzed with Agilent Masshunter Quantitative Analysis (Version B.08.00).

### 2.3. Statistics

For this secondary analysis, the subsets of Nx rats (n = 22) and sham rats (n = 18) randomized to the high P or low P diets were included in the analysis. Sample size in the parent study was calculated based on phosphorus absorption data in Nx from Marks et al. [22,26]. Statistics were completed using Statistical Analysis Software version 9.4 (SAS Institute, Raleigh, NC, USA). Two-way ANOVA was performed for all outcomes of this ancillary study, with main effects for health status, diet, and their interaction compared using Tukey post hoc comparisons; statistical significance was set at α < 0.05. Model residuals were normally distributed for most comparisons. When model residuals were not normally distributed, data were transformed using logarithmic transformations prior to statistical analysis. When logarithmic transformations were performed, the data were normalized and the equality of variances assumption was satisfied. Back-transformed values are presented in their original units. All data are presented as mean ± SEM of the original data, unless otherwise specified. *p*-values are presented for the overall two-way ANOVA model (p_model_), Nx vs. sham (p_health status_), high P vs. low P diet (p_diet_), and the interaction between the two model variables (i.e., interaction between health status and diet, p_interaction_).

## 3. Results

### 3.1. Model Verification and Food Consumption

**Figure 2:**
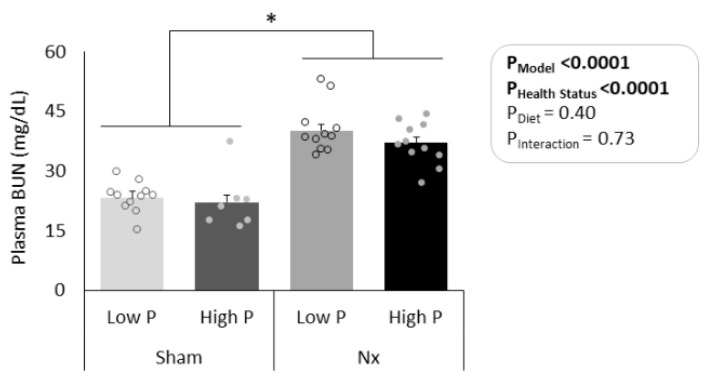
Plasma blood urea nitrogen (BUN) at sacrifice (n = 7–11/group). Nx rats had significantly elevated plasma BUN levels, indicating reduced kidney function. * *p* < 0.0001. Nx = 5/6th nephrectomized rats; P = phosphorus. Health status refers to sham vs. Nx. Data shown as mean ± SEM.

**Figure 3:**
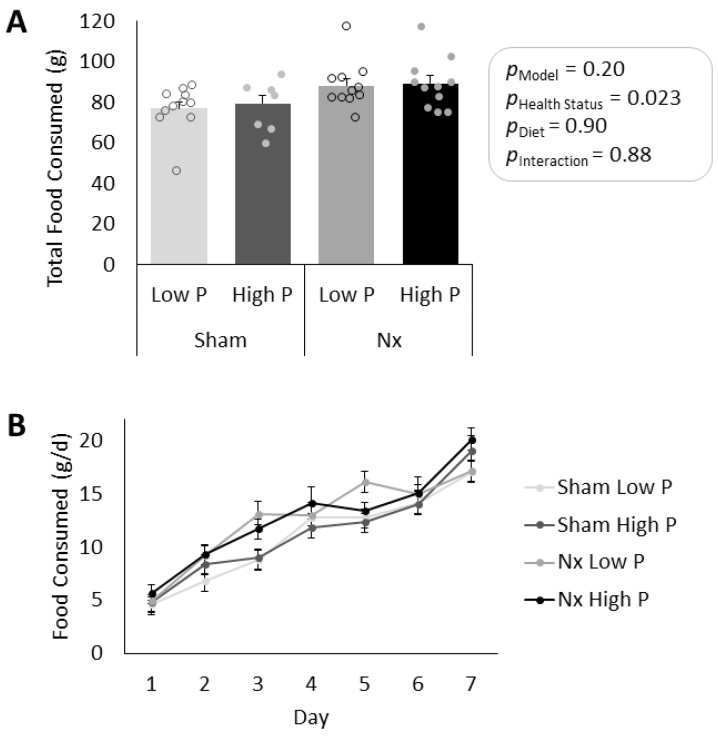
Food consumption during 1 week low P (0.1% (*w*/*w*)) or high P (1.2% (*w*/*w*)) diets. (**A**) Total food consumption over 7d period on test diet. There were no significant differences between groups (*p*-values shown in figure). (**B**) Daily food consumption during 4 h/d time-restricted feeding for each day of study. Rats in all groups consumed more food as the intervention period progressed. Nx = 5/6th nephrectomized rats; P = phosphorus. Health status refers to sham vs. Nx. Data shown as mean ± SEM.

In all sham rats, both kidneys were intact and appeared normal at the time of sacrifice. In all Nx rats, only the middle third of the left kidney was present and appeared enlarged at sacrifice. As expected, plasma BUN was significantly elevated in Nx rats as compared to sham controls (*p* < 0.0001, Figure 2), and BUN values were similar to values previously observed for both sham and Nx male rats at a similar age and post-operative time [27]. There were no differences in BUN due to diet (*p* = 0.40) or interaction effects (*p* = 0.73). Total food consumption over 7d was similar between all treatment groups (p_model_ = 0.20), though rats in all groups increased their daily intake over the 7 days on the diets (Figure 3). The increase in food consumption was likely due to the switch from ad libitum feeding to time-restricted feeding during the study period, which was part of the experimental design of the parent study [22].

### 3.2. Effects of Dietary Phosphorus Intake and CKD on Serum UT 

**Figure 4:**
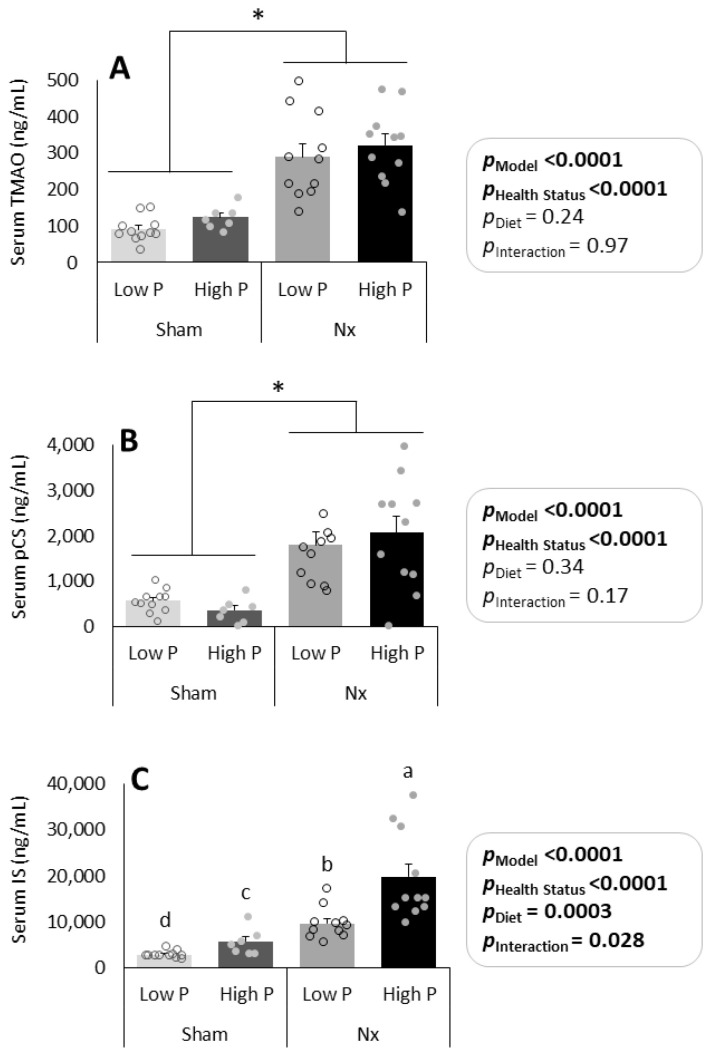
Serum UT for all rats (n = 7–11/gp) at sacrifice. TMAO (**A**), pCS (**B**), and IS (**C**) were significantly higher in Nx vs. sham rats, though dietary phosphorus level only affected IS levels (*p*-values shown in figure). * *p* < 0.0001; lowercase letters indicate significant differences between treatment groups compared to each other (*p* < 0.05). TMAO = trimethylamine oxide; IS = indoxyl sulfate; pCS = p-cresol sulfate; Nx = 5/6th nephrectomized rats; P = phosphorus. Health status refers to sham vs. Nx. Data shown as mean ± SEM.

Serum TMAO was significantly higher in Nx than in sham operated rats (*p* < 0.0001), though there were no differences due to dietary phosphorus level (*p* = 0.24) or interaction effects (*p* = 0.97) (Figure 4A). Similarly, serum pCS was significantly higher in Nx compared with sham operated rats (*p* < 0.0001), but there were no differences due to dietary phosphorus level (*p* = 0.34) or interaction effects (*p* = 0.17) (Figure 4B).

Serum IS was significantly different among all groups at sacrifice (Figure 4C). Rats on the high P diet had higher serum IS than low P rats (*p* = 0.0003), and Nx rats had higher levels than sham operated rats (*p* < 0.0001). However, there was also a significant interaction effect between diet and CKD status (*p* = 0.028), where the diet effect was of a greater magnitude in Nx rats than in sham rats. 

### 3.3. Effects of a Change from Baseline Diet Macronutrient Composition and Source on Serum UT 

**Figure 5:**
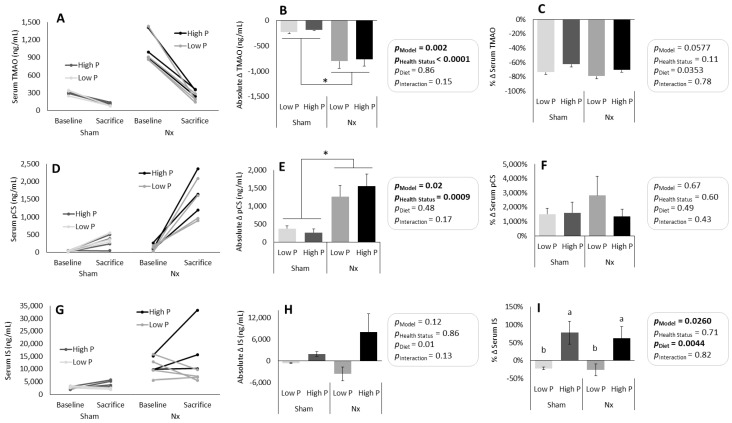
Change in serum UT in a subset of rats (n = 3–4/gp) from baseline to sacrifice. TMAO decreased in all rats (**A**), with the absolute change being larger in Nx than sham rats (**B**), though the relative change was the same across all treatment groups (**C**). pCS increased in all rats (**D**), with the absolute change being larger in Nx than sham rats (**E**), though the relative change was not significantly different across treatment groups (**F**). IS increased in rats on the high P diet but decreased in rats on the low P diet (**G**,**H**), regardless of health status; the relative change in IS was significantly different based on dietary phosphorus level (**I**). Lowercase letters indicate significant differences between treatment groups (*p* < 0.05). * *p* < 0.001. TMAO = trimethylamine oxide; IS = indoxyl sulfate; pCS = p-cresol sulfate; Nx = 5/6th nephrectomized rats; P = phosphorus. Health status refers to sham vs. Nx. Data shown as mean ± SEM.

To further investigate the changes in UT and to see if there was a change in UT due to the switch from Teklad 2018 to low P and high P diets, we explored changes in serum UT levels from baseline to sacrifice in a subset of rats (n = 3–4/group). Serum UT were higher in Nx rats as compared to sham rats both before and after the 7d on experimental high P and low P diets, but the direction and magnitude of change from Teklad 2018 to low P and high P diets varied between UT (Figure 5).

Serum TMAO levels decreased in all rats during the 7d experimental diet, regardless of dietary phosphorus content or health status (Figure 5A). The absolute change was significantly greater in the Nx as compared to the sham rats (Figure 5B). When considering the relative change in circulating TMAO levels (Figure 5C), there was a tendency towards a larger percentage decrease in TMAO on the low P diet as compared to the high P diet (p_Diet_ = 0.0353), though the overall model did not show a statistically significant difference between groups (p_Model_ = 0.0577).

Serum pCS levels increased in all rats during the 7d experimental diet, regardless of dietary phosphorus content or health status (Figure 5D). The absolute change in serum pCS was significantly greater in Nx as compared to sham rats (*p* = 0.02, Figure 5E), though the relative change showed no significant differences between groups (*p* = 0.67, Figure 5F).

Serum IS levels increased in some rats and decreased in others, regardless of health status (Figure 5G). There were no significant differences between groups in the absolute change in serum IS (Figure 5H), but there were significant differences in the relative change between groups, with rats on the high P diet having significantly higher serum IS levels than rats on the low P diet (Figure 5I).

## 4. Discussion

In this secondary analysis, we investigated the effect of high versus low dietary phosphorus on serum UT levels in Nx and sham operated rats. As expected, Nx rats had significantly higher levels of serum UT than sham-operated rats. This is consistent with the prior literature indicating that declining kidney function leads to an accumulation of circulating levels of UT [28,29]. However, when investigating the effect of dietary phosphorus levels on the production of UT, our results suggest that different dietary phosphorus levels significantly affect serum IS but not serum TMAO or pCS (Figure 4). Because the UT evaluated in this study are derived from amino acid fermentation, we further explored changes in serum UT from baseline to sacrifice in a subset of rats (Figure 5). At baseline, rats had been on the grain/soy-based Teklad 2018 diet (protein sources: wheat, corn, and soybeans), whereas, at sacrifice, they had been switched for 1 week onto the egg white/sucrose-based low P and high P diets (protein source: egg white solids). The TMAO precursors (betaine, choline, and L-carnitine) were present in higher amounts in the Teklad 2018 diet than in the low P and high P diets, while the IS and pCS precursors (tryptophan and tyrosine & phenylalanine, respectively) were present at similar levels in all diets [30,31,32]. As discussed below, these changes in the dietary protein source are consistent with the direction of change observed from baseline to sacrifice for TMAO and IS (but not pCS) production during the study, indicating that the protein source influences UT production.

Phosphorus is an essential nutrient for all living organisms, including microorganisms that reside in the gut microbiome. However, there are limited data on the potential effects of different dietary phosphorus loads on the gut microbiome and the subsequent production of UT. A recent study [21] tested the effects of 4 weeks on a diet with normal (0.8%) vs. high (1.2%) phosphorus in sham and Nx rats on the gut microbiota, using the same animal model as the current study. Rats receiving the high-phosphorus diet had higher relative abundance of the genera Blautia, Allobaculum, and unclassified Clostridiales compared with rats fed a normal phosphorus diet. Some members of the unclassified Clostridiales and Allobaculum can metabolize tryptophan into indoles, which are precursors to IS [33,34]. While we did not measure fecal microbiota composition in our study, the microbial changes reported by Ye et al. [21] and our results suggest that high dietary phosphorus may lead to increased relative abundance of indole-producing bacteria and circulating levels of indole-derived UT, including IS. Despite the findings of this study and others, there are still limited data on the potential effects of different dietary phosphorus levels on the gut microbiome, especially in CKD, which provides a unique area for future research.

### Strengths and Limitations

Rodents and humans have differences in the gastrointestinal tract anatomy that may impact the generation of microbially derived UT. Notably, the large intestine is different. Rodents are cecal fermenters, whereas in humans most of the microbial fermentation occurs in the colon. Furthermore, the microbially derived precursors of UT (i.e., indole, phenol) undergo phase I (e.g., oxidation) and II metabolism (e.g., sulfation). To our knowledge, enzymatic reactions to generate microbially derived UT are similar in rodents versus in humans.

Our study has several strengths and limitations. This was a controlled feeding study using Nx rats, which are a commonly used animal model to study CKD. By controlling the diet, we were able to compare the effects of both low- and high-phosphorus diets on UT levels. The major limitations of this study were the short intervention period, lack of gut microbiome information, and the limited availability of baseline samples from the parent study.

## 5. Conclusions

In conclusion, in this ancillary, exploratory study we found that different dietary phosphorus levels altered serum levels of IS, but not TMAO or pCS, in a rat model of CKD. To our knowledge, this is the first study to demonstrate the effect of different dietary phosphorus levels on the production of UT. Our results suggest that restricting dietary phosphorus in CKD patients may also reduce circulating levels of IS. Because higher IS levels are associated with adverse outcomes in CKD patients, our results lend support to the current recommendation for CKD patients to avoid high phosphorus intakes. Future studies investigating the link between dietary phosphorus, gut microbiota, and UT production in CKD patients are necessary to verify our preclinical results. Additional studies to establish the potential mechanistic links between dietary phosphorus, protein, and fiber and UT production in CKD are also warranted.

## Data Availability

The data presented in this study are available in the article and Supplementary Materials.

## Supplementary Materials

The following supporting information can be downloaded at https://www.mdpi.com/article/10.3390/nu16121807/s1: Table S1: Comparison of diet composition; Table S2: LP and HP diet formulations; Table S3: Multiple reaction monitoring table for data acquisition.
