# Supplementary material for: Dietary Phosphorus Levels Influence Protein-Derived Uremic Toxin Production in Nephrectomized Male Rats
Source: Nutrients. 2024 Jun 8;16(12):1807. doi: 10.3390/nu16121807 (PMC11207110; doi:10.3390/nu16121807)
Supplement: Supplementary file 1 [file nutrients-16-01807-s001.zip › nutrients-3043899-supplementary.pdf]

Table S1. Comparison of diet composition.

	Teklad 2018 Diet	LP diet (TD.85010)	HP diet (TD.85349)
Kcal/g	3.1	3.8	3.7
Protein (% kcal from)	24	17.2	17.6
Carbohydrate (% kcal from)	58	70.8	70.1
Fat (% kcal from)	18	12	12.3
Total Phosphorus (%)	0.7	0.1	1.2
Non-phytate phosphorus (%)	0.4	0.1	1.2

Table S2. LP and HP diet formulations.*

	LP diet (TD.85010) (g/kg)	HP diet (TD.85349) (g/kg)
Egg White Solids	200	200
Sucrose	525.2	501.2
Corn Starch	150	150
Corn Oil	50	50
Cellulose	20	20
Mineral Mix, Ca-P Deficient (79055)	13.4	13.4
Calcium Carbonate	14.7	5.03
Potassium Bicarbonate	8.1	-
Sodium Chloride	4.7	-
Sodium Phosphate, Monobasic, Monohydrate	1.7	12.9
Potassium Phosphate, Monobasic	1.7	12.7
Calcium Phosphate, Monobasic, Monohydrate	-	24.4
Vitamin Mix, Teklad (40060)	10	10
Biotin	0.004	0.004
Yellow Food Color	0.3	0.3
Blue Food Color	0.15	-
Red Food Color	-	0.15

*The complete formulation of Teklad 2018 diet is available at https://www.envigo.com/rodent-natural-ingredient-2018-diets.

Table S3. Multiple reaction monitoring table for data acquisition.

Compound Name	Precursor Ion (m/z)	Product Ion (m/z)	Collision Energy (V)	ESI Polarity
3-indoxyl sulfate-d4	216	80.9	10	Negative
3-indoxyl sulfate-d4	216	79.9	20	Negative
3-indoxyl sulfate-d4	216	136	15	Negative
3-indoxyl sulfate	212	132	15	Negative
3-indoxyl sulfate	212	80.9	10	Negative
3-indoxyl sulfate	212	79.9	20	Negative
p-cresol sulfate-d7	194	114	20	Negative
p-cresol sulfate-d7	194	79.9	30	Negative
p-cresol sulfate	187	107	20	Negative
p-cresol sulfate	187	79.9	30	Negative
TMAO-d9	85.1	68.1	10	Positive
TMAO-d9	85.1	66.1	20	Positive
TMAO-d9	85.1	46.1	45	Positive
TMAO	76.1	59.1	10	Positive
TMAO	76.1	58.1	20	Positive
TMAO	76.1	42.1	45	Positive
